# Impact of air pollution on exacerbations of respiratory and cardiovascular diseases for the intensivist: a narrative review

**DOI:** 10.1186/s40635-026-00908-2

**Published:** 2026-05-13

**Authors:** Alexandre Gaudet, Charlotte Lacombe, Gaetan Piga, Patricia de Nadaï, Group for the Assessment of ICU-related Impacts of Air pollution (GAÏA)

**Affiliations:** 1 CHU Lille, Intensive Care Medicine Department, Roger Salengro Hospital, CHU Lille, 59000 Lille, France; 2 Univ. Lille, CNRS, Inserm, CHU Lille, Institut Pasteur de Lille, U1019-UMR9017-CIIL-Center for Infection and Immunity of Lille, 59000 Lille, France; 3 Group for the Assessment of ICU-related Impacts of Air Pollution (GAÏA), Lille, France

**Keywords:** Air pollution, Airborne particulate matter, Intensive care, Respiratory diseases, Cardiovascular diseases

## Abstract

Air pollution is a major global health threat, yet its specific impact on critically ill patients remains underexplored. While the link between air pollution and respiratory and cardiovascular events is well-documented, its contribution to organ dysfunction in the ICU setting requires a comprehensive synthesis for the intensivist. This narrative review aims to summarize the pathophysiological mechanisms and epidemiological evidence linking short-term and long-term air pollution exposure to exacerbations of respiratory and cardiovascular diseases, with a focus on critical illness. We therefore conducted a comprehensive review of the literature, focusing on clinical studies and mechanistic models. We specifically examined the impact of particulate matter and gaseous pollutants on ICU-specific outcomes. Evidence indicates that air pollution notably acts as a potent trigger for acute respiratory distress syndrome, acute lower respiratory infections, and exacerbations of chronic obstructive pulmonary disease and asthma. Higher levels of air pollution are associated with acute cardiovascular events leading to hospital admission, especially myocardial infarction, stroke, acute heart failure, and arrhythmia. Recent data suggest that long-term exposure significantly increases the susceptibility to critical illness, while short-term peaks are associated with immediate spikes in ICU mortality and morbidity. In conclusion, air pollution is a silent but significant contributor to the burden of critical illness. For the intensivist, understanding these environmental determinants is crucial for both clinical risk assessment and public health advocacy. Future research must bridge the gap between general population observations and direct clinical evidence in the ICU to refine protective strategies in the context of a changing climate.

**Graphical abstract:**

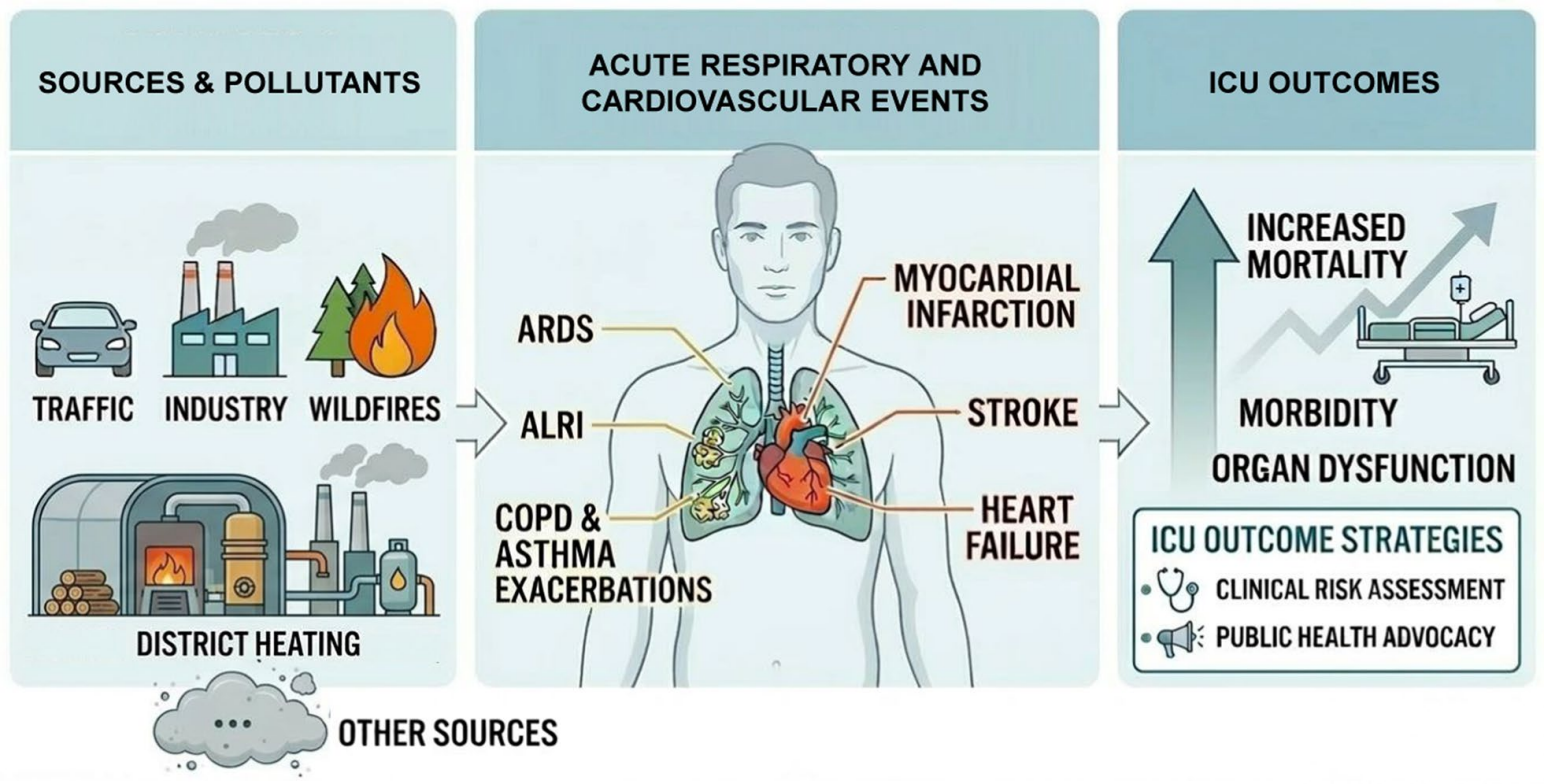

## Introduction

Exposure to air pollutants and its impact on human health are growing concerns within society. The influence of environmental conditions on the incidence and severity of certain respiratory and cardiovascular conditions has been highlighted by numerous epidemiological and pathophysiological studies. However, the effects of air pollution on critically ill patients remain relatively unknown.

In this review, we aim to synthesize the available literature to better understand the challenges related to air pollution—sometimes linked to climate change—on critically ill patients.

## Air pollution

### Definition and composition

Air pollution is generally defined as the human-driven introduction of substances or energy into the environment that results in harmful effects on health, ecosystems, and infrastructure [1].

A broader biological perspective defines a pollutant as an extrinsic agent that, above a specific threshold, induces negative impacts on cellular or environmental integrity [2].

**Fig. 1:**
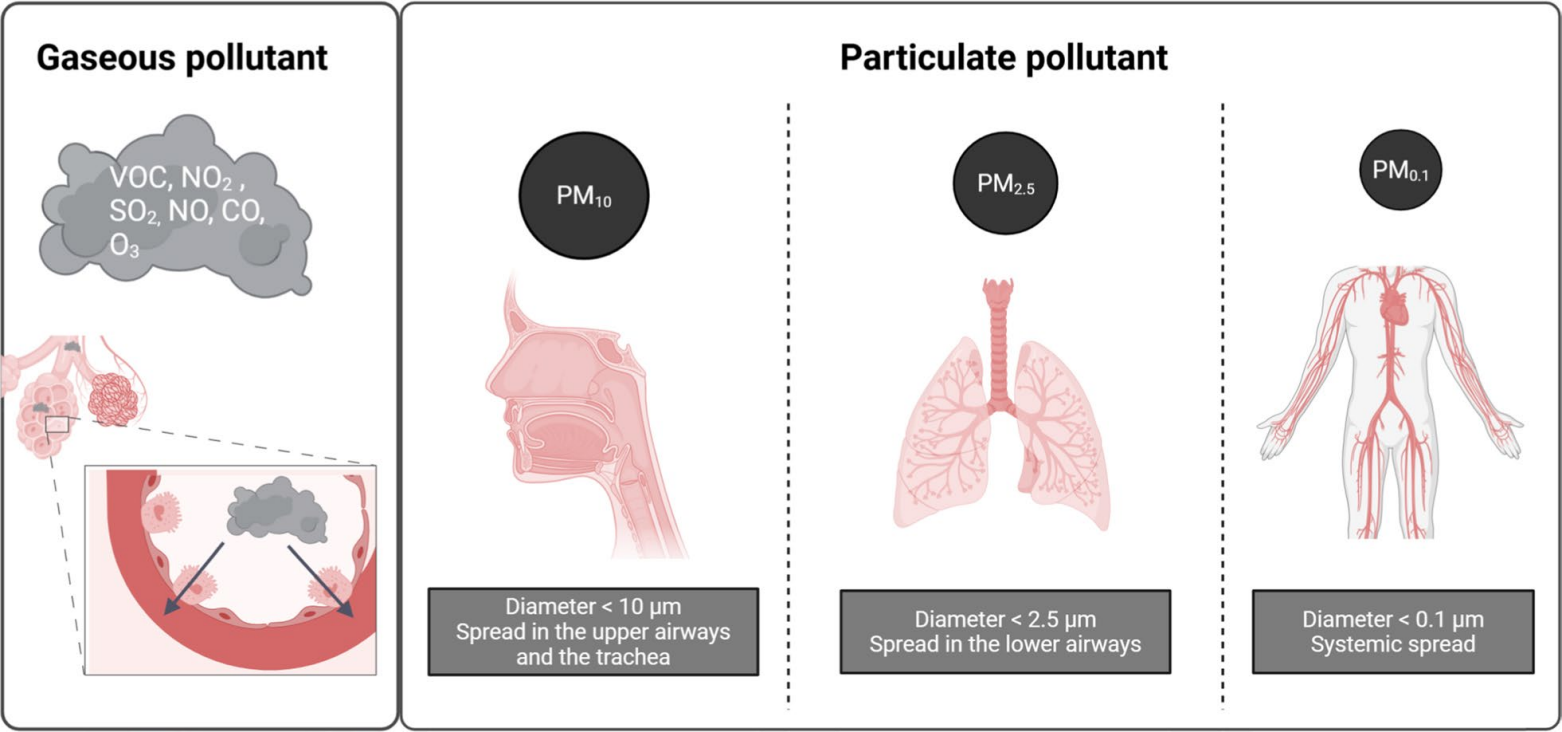
The different types of atmospheric pollutants. There are two categories of atmospheric pollutants: gaseous pollutants and particulate pollutants. Gaseous pollutants are inhaled and then diffuse into the respiratory system until they reach the alveoli. Particulate pollutants are classified based on their size. Organ diffusion depends on the size of the particles. VOCs, volatile organic compounds, NO_2_, nitrogen dioxide; SO_2_, sulfur dioxide; NO, nitrogen monoxide; CO, carbon monoxide; O_3_, ozone; PM, particulate matter

Beyond its primary components (N_2_ and O_2_), air contains a complex mixture of anthropogenic and natural toxins divided into two phases:

- Gaseous phase: Includes O_3_, NO_2_, SO_2_, NH_3_, CO, volatile organic compounds (VOCs), and polycyclic aromatic hydrocarbons (PAHs).
- Particulate phase: Comprises heavy metals and particulate matter (PM) [3], a suspension of solid and liquid compounds classified by aerodynamic diameter [4], which determines their respiratory penetration (Fig. 1):
  o PM_10_ (coarse particles): <10 μm; reach the upper airways and trachea.
  o PM_2.5_ (fine particles): <2.5 μm; penetrate the lower airways.
  o PM_0.1_ (ultrafine particles/UFPs): <0.1 μm; can disseminate into systemic circulation.

While NO_2_, SO_2_, O_3_, PM_10_, and PM_2.5_ are routinely monitored for epidemiological studies, PM_0.1_ concentrations are not, leading to significant data gaps regarding their impact [5].

### Sources and atmospheric dynamics

Pollutants originate from diverse human activities:

- Road transport: a primary source of NO_2_ and diesel exhaust particles (DEP), though stricter standards are reducing their PM_2.5_ contribution.
- Industry and heating: the predominant sources of PM_2.5_ and SO_2_.
- Secondary formation: O_3_ is not emitted directly but formed by the reaction of precursors (like NO_2_) under solar radiation [6].

Ambient air quality at any location results from a synergy of local emissions, long-range transport, and meteorological factors (wind, rain, temperature, and UV radiation) that govern the dispersion, resuspension, and chemical transformation of pollutants [7].

Finally, indoor pollution significantly contributes to overall exposure, arising from building materials, combustion appliances, and lifestyle factors such as smoking or the presence of pets [8].

## Impact of environmental conditions on air pollution

The evolution of air quality is deeply intertwined with climate change, suggesting a significant worsening of pollutant exposure in the coming decades.

A strong causal link exists between extreme heat and air quality, with particulate matter and ozone concentrations rising sharply during heatwaves [9–11]. Given Intergovernmental Panel on Climate Change (IPCC) projections of more frequent and intense heatwaves, this relationship poses a growing public health challenge [12].

Wildfires represent a major, yet often underestimated, source of PM_2.5_. A study of 140 million hospitalizations in Brazil found that every 10 µg/m^3^ increase in wildfire-related PM_2.5_ raised respiratory admissions by 5% within 24 h [13]. These pollutants travel vast distances: smoke from the 2022 French Arcachon fires reached the Belgian border (40 µg/m^3^), while 2020 U.S. West Coast fires produced a pollution cloud that crossed the Atlantic to Europe in 6 days [14, 15]. Burned areas have doubled in the U.S. recently (2002–2020 vs. 1984–2001), a trend mirrored in Europe where the geography of fires is shifting northward [16–18]. Non-Mediterranean regions, which accounted for only 10% of European burned areas in 2006–2013, represented 50% in 2022. This shift is driven by rising temperatures and increasingly frequent droughts [18, 19].

Finally, the expansion of the Sahara has led to a progressive increase in sandstorm-related particulate pollution in Western Europe. According to the Copernicus Observatory, these events are expected to grow in frequency and intensity due to climate change [20].

## Impact on acute conditions

### Pathophysiological consequences

#### Acute lung inflammatory response

The literature has also aimed to clarify the pathophysiological aspects of the impact of exposure to atmospheric pollutants on health, primarily focusing on pulmonary inflammation.

O_3_ impairs innate immune function (macrophage/neutrophil killing), barrier integrity, and mucociliary clearance, providing mechanistic underpinnings for increased infection susceptibility [21–24]. Models combining exposures to O_3_ and LPS reproduce key features of acute respiratory distress syndrome (ARDS), including increased BAL protein, sRAGE, and neutrophilic consolidations [25–28]. PM-focused data similarly highlight impaired host defense, increased pneumonia risk, and early ALI/ARDS-like inflammation after high or sustained exposures [29, 30].

However, the local inflammatory response appears to differ depending on the mode and timing of pollutant exposure. Experimental data in rats show that high-dose petroleum combustion particles induce acute oxidative pulmonary inflammation within 24 h [31], a result recently replicated in mice exposed for 3 weeks to lower, Sidney city-level PM_10_ doses [32]. Regarding the exudative-edematous phase of acute respiratory distress syndrome (ARDS), high-dose DEP concurrently administered with LPS exacerbate neutrophilic infiltrates and ARDS-like features up to 48 h post-LPS exposure [33–35]. Conversely, divergent results emerge with prolonged PM_2.5_ exposure. Five weeks of PM_2.5_ prior to LPS pulmonary instillation can actually attenuate the acute inflammatory response, reducing neutrophilic infiltrates and pro-inflammatory cytokines expression [36]. Yet, when this prolonged daily exposure to PM_2.5_ is initiated after the onset of acute pulmonary inflammation, it leads to increased late-phase neutrophilic infiltrates, heterogeneous emphysematous changes and altered collagen/elastic fiber composition [37]. However, to our knowledge, no data exist on the physiological impact of these lesions in terms of gas exchange or ventilatory mechanics. These data collectively support the concept that ambient pollutant constitute a modifiable “priming” factor that can shift a given infectious or inflammatory insult toward ARDS, particularly in vulnerable hosts.

Regarding allergic and asthma-related mechanisms, human exposure studies demonstrate that O_3_ doses of 100–200 ppb increase airway neutrophilia and alter BAL cell transcriptional profiles; notably, asthmatic subjects often exhibit more pronounced pathway activation (inflammation, repair) than healthy controls [24, 38–40]. In allergic models, acute O₃ exposure further exacerbates airway hyperresponsiveness, TNF-α, IL-13, hyaluronan, and mucus (Muc5ac) production, while inducing epithelial desquamation [41]. PM_2.5_ and PM_0.1_ enhance allergen-induced aryl hydrocarbon receptor (AhR) signaling and eosinophilic inflammation. In both human cohorts and animal models, these effects are mediated in part via epithelial IL-25 and IL-33, as well as M2 macrophage polarization and eotaxin-1 production [42, 43]. In vitro, human bronchial epithelium exposed to PM_2.5_ upregulates IL-6/IL-8 and COX-2 through ROS–JAK2/STAT3 pathway [44]; similarly, nasal epithelial spheroids from asthmatics exposed to 100 µg/ml PM_2.5_ for 24 h produce significantly higher levels of IL-8 and TNFα than those from healthy controls [45]. Collectively, these data support a model wherein pollutants prime or amplify ongoing type-2 and neutrophilic pathways, lower the threshold for airway hyperreactivity, and intensify responses to allergens and infections.

Similarly, the link between air pollution and COPD has been extensively investigated. Chronic O₃ exposure in mice recapitulates features of COPD (including airway inflammation, hyperreactivity, and emphysema), driven by mitochondrial ROS and NLRP3 inflammasome activation. Notably, mitochondrial antioxidants and caspase-1 inhibition have been shown to attenuate both acute inflammation and chronic structural changes [46–48]. Human COPD airway smooth muscle cells exhibit baseline mitochondrial dysfunction; O3-mediated oxidative stress exacerbates this impairment, which is partly reversible with mitochondria-targeted antioxidants [47]. Human and experimental evidence further demonstrates that PM_2.5_ triggers acute COPD exacerbations by amplifying neutrophilic and systemic inflammation, increasing oxidative stress markers, and impairing macrophage phagocytosis and efferocytosis. These pollutants also modify the airway microbiota and coagulation markers [49]. Even at doses approaching environmental levels, low-dose traffic PM_10_ elevates BAL macrophages and lymphocytes, activates NLRP3, and depletes mitochondrial antioxidants. Furthermore, the COPD epithelium is particularly vulnerable to PM-induced tight junction loss and microbial colonization [50–52]. Overall, these findings illustrate how exposure to O₃ and PM translate into clinically recognized COPD exacerbations via a rapid surge in oxidative load, neutrophilic inflammation, mucus hypersecretion, and structural small-airway compromise.

#### Consequences on the quality of the immune response

Beyond the severity of the inflammatory reaction, several studies have explored the qualitative effects of pollution on the immune response in humans and animals.

Air pollutants act as profound modulators of the respiratory environment by reshaping the protease landscape and compromising frontline defenses. For RNA viruses like influenza and SARS-CoV-2, O_3_ exposure facilitates entry by increasing airway protease activity and reducing the antiprotease SLPI, thereby shifting the balance toward the cleavage and activation of viral hemagglutinin [53]. Beyond these structural alterations, pollutants accelerate viral binding and penetration through the upregulation of adhesion molecules such as ICAM-1 and PAFR, while particulate matter (PM) can act as a physical carrier, forming virus-pollutant conjugates that prolong viral survival and enhance epithelial access [54–58]. This impairment of innate immune function, barrier integrity, and mucociliary clearance (MCC) provides the mechanistic underpinnings for increased infection susceptibility observed across multiple models [21–24]. Crucially, ambient PM suppresses core antiviral circuits, including type I IFN production and IRF3 activation, creating a permissive environment for replication even when pro-inflammatory cytokine output remains high [59–63]. This shift toward an impaired immune phenotype is further supported by experimental models, where exposure to NO_2_ induced a pulmonary cytokine profile shift toward a Th2 phenotype, manifesting as a 50% reduction in pulmonary IFNγ expression [64]. Clinical evidence in healthy volunteers has similarly demonstrated a weakened IFNγ response following ex vivo lymphocyte stimulation during periods of higher pollutant exposure compared to lockdown periods. This systemic blunting of the immune response may directly promote severe clinical outcomes, as evidenced by the increased risk of SARS-CoV-2 hospitalization identified in subjects with abnormally low baseline IFNγ expression [64].

The impact of pollution extends to bacterial susceptibility by paralyzing macrophage and neutrophil functions. PM exposure impairs the phagocytic and bactericidal signaling of alveolar macrophages, specifically targeting internalization pathways and suppressing PI3K/Akt and NFκB signaling [65–70].

Furthermore, pollutants drive the expression of immune checkpoint molecules such as PD-L1 on neutrophils, which directly compromises antibacterial defense [71]. Recent murine studies have identified a novel SiglecF + neutrophil population in bronchoalveolar lavage following diesel exhaust particle (DEP) exposure; these cells, which were absent in LPS-only models, constitute nearly 30% of total lavage cells and exhibit an increased capacity for NETosis [72]. Although not yet studied in humans, this pathway represents a promising avenue for identifying specific patient phenotypes whose organ dysfunction could be triggered or worsened by air pollution.

**Fig. 2:**
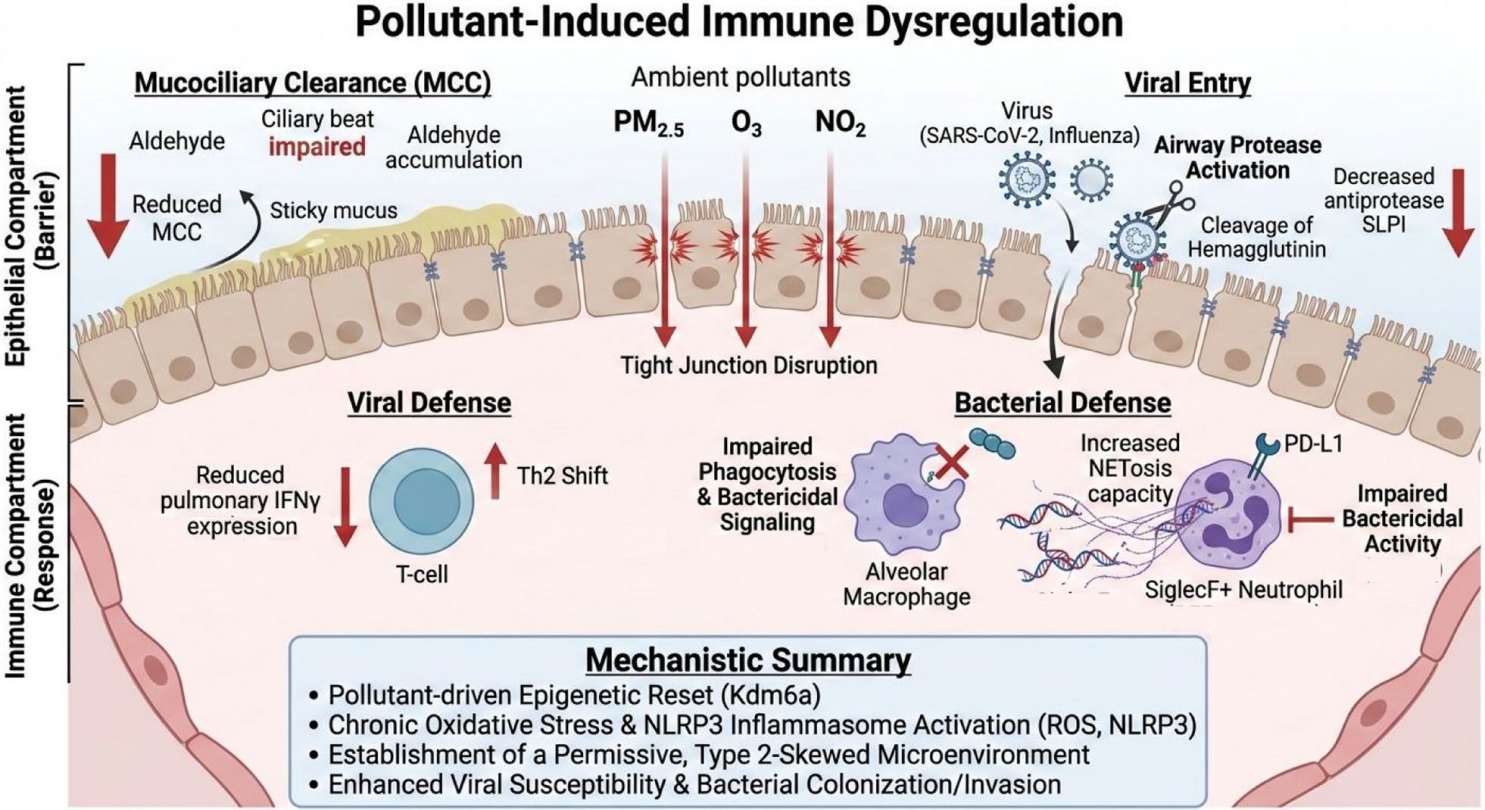
Impact of air pollution on respiratory immune dysregulation. Ambient air pollutants (PM_2.5_, O_3_, NO_2_) dismantle respiratory defenses across three interrelated compartments. In the epithelial compartment, pollutants directly disrupt tight junctions and cause ciliary beat paralysis due to the accumulation of reactive aldehydes, leading to severely impaired mucociliary clearance. Simultaneously, O_3_ exposure enhances viral entry by increasing airway protease activation, which cleaves and activates viral hemagglutinin, an effect compounded by a decrease in SLPI, an antiprotease which protects against excessive inflammatory immune responses at epithelial barriers. Within the immune compartment, chronic pollution primes the host for infection. Regarding viral defense, lymphocytes exhibit a reduction in IFNγ expression and a concurrent Th2 shift, weakening the primary antiviral response. Regarding bacterial defense, pollutants paralyze innate cellular defense; alveolar macrophages show impaired phagocytosis, and neutrophils are reprogrammed to express high levels of the checkpoint molecule PD-L1, inhibiting bactericidal function. Furthermore, DEPs recruit a specific SiglecF + neutrophil population with an increased capacity for NETosis, contributing to lung injury. These collective alterations—including an epigenetic reset via Kdm6a and chronic oxidative stress—establish a permissive, Th2-skewed microenvironment that enhances vulnerability to subsequent infectious or inflammatory insults. DEP, Diesel exhaust particle; MCC, mucociliary clearance; NO_2_, nitrogen dioxide; O_3_, ozone; PM, particulate matter; ROS, reactive oxygen species

Finally, pollution undermines epithelial integrity by reducing antimicrobial peptides like human β-defensin 1 and saturating detoxification enzymes such as ALDH1A1, leading to the accumulation of reactive aldehydes that selectively paralyze mucociliary clearance [73, 74]. By reshaping the respiratory microbiota—increasing the prevalence of genera such as Streptococcus and Prevotella—and acting as a "Trojan horse" for pathogen delivery, ambient particles fundamentally reset the host–pathogen interface [67, 75]. These collective data, including transcriptomic and epigenetic analyses, support a model where chronic pollutant exposure can "reset" macrophage and epithelial responses, potentially contributing to sustained susceptibility or worsened outcomes during successive infectious seasons [58, 60, 76–78] (Fig. 2).

#### Impacts on the cardiovascular system

Extensive literature highlights the role of air pollution in increasing the risk of cardiovascular event. Several mechanisms have been proposed to explain this association. Exposure to air pollutants is responsible for oxidative stress and systemic inflammation. In particular, particle-bound redox-active species and the surface area of ultrafine particles generate reactive oxygen species in the vasculature and alter key enzyme systems: NADPH oxidases, the mitochondrial respiratory chain, uncoupled eNOS, and xanthine oxidase. Consequently, exposure to air pollutants is associated with an increase in circulating IL-6, TNF-α, CRP, and fibrinogen, as well as altered lipid oxidation and the priming of platelets and endothelial cells, creating a pro-thrombotic, pro-atherogenic milieu [82–84].

In addition, exposure of healthy individuals to particulate pollution appears to induce a rapid impairment of endothelial function, especially reducing nitric oxide (NO) synthesis and possibly increasing endothelin production, leading to elevated diastolic blood pressure [85–88]. Furthermore, the 2024 meta‑analysis by Wang et al*.* shows that short‑term increases in PM_2.5_ raise soluble ET‑1, E‑selectin, ICAM‑1, VCAM‑1, quantitatively supporting the hypothesis of an activated, pro‑adhesive endothelium [89].

In addition, multiple studies have demonstrated the thrombotic effect of particulate pollution, particularly diesel particles, by increasing erythrocyte adhesiveness and decreasing fibrinolytic activity in the vascular compartment [87, 90, 91]. Along the same lines, PM_2.5_ acutely shifts hemostasis toward thrombosis through platelet activation (marked by increased P-selectin and aggregation), elevated tissue factor and thrombin levels, and impaired fibrinolysis (characterized by increased PAI-1 and decreased t-PA) [92]. The pro-inflammatory effect of particulate pollutants also seems to correlate with the progression of atherosclerotic lesions in animal models [93]. This was further supported by Rao et al*.*, who showed in mice that chronic air pollution exposure drives CD36-dependent 7-ketocholesterol accumulation in macrophages, accelerating atherosclerosis—one of the clearest molecular links from inhaled PM to atheroma plaque biology [94].

Finally, time‑series and panel studies show short‑term PM_2.5_/traffic exposure to be associated with reduced heart rate variability, increased heart rate, and changes in repolarization (QT, T‑wave parameters), yielding a substrate for arrhythmias [95]. Mechanisms likely involve effects on ion channels and action potential duration [96]. This links air pollution to atrial fibrillation and ventricular arrhythmias, and finally to triggering of sudden cardiac death in susceptible individuals.

Collectively, these findings suggest a strong link between exposure to air pollutants and the activation of pathophysiological pathways associated with acute cardiovascular events.

### Epidemiological aspects

**Fig. 3:**
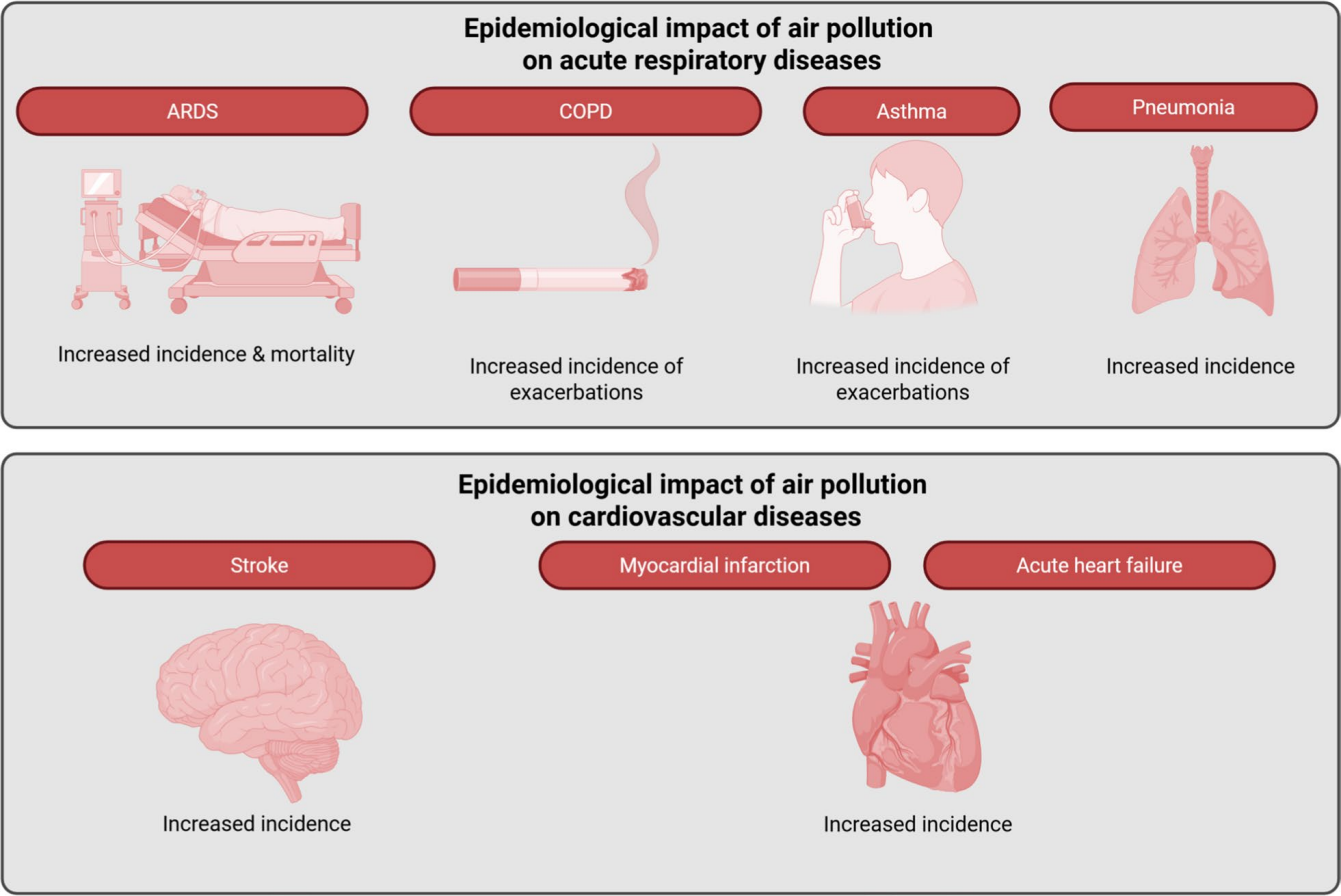
Exposure to air pollutions—Impact on the epidemiology of acute pulmonary and cardiovascular diseases. Exposure to air pollution has an impact on the epidemiology of many acute diseases, particularly respiratory and cardiovascular diseases. Long-term and short-term exposures to air pollution are associated with an increase in the incidence of ARDS, COPD and asthma exacerbations, pneumonia, stroke, myocardial infarction and acute heart failure. In critically ill patients, long-term exposure to air pollution is associated with longer duration of mechanical ventilation and increased mortality in patients admitted for cardiopulmonary diseases and ARDS. ARDS, acute respiratory distress syndrome; COPD, chronic obstructive pulmonary disease

The question of the impact of pollution on the occurrence of acute diseases actually encompasses two different facets of a broader problem. On the one hand, the impact of acute exposure, of short duration, typically lasting a few days and generally less than a week, to much higher levels, which can reach several hundred µg/m^3^ for PM_2.5_. On the other hand, the effects of chronic exposure, lasting from several months to years, to “moderate” levels, generally around 10–20 µg/m^3^ for PM_2.5_ in European metropolitan areas. While these different exposure modalities, distinct in their nature, both appear to have a real impact on the occurrence of acute pathologies, some of their characteristics lead to their analysis through different prisms (Fig. 3).

#### Short-term exposure

##### Cardiovascular admissions

The effects of pollution peaks on the incidence and severity of acute pathologies have been widely studied. The North American SPARCS database allowed the analysis of a total of 2,000,000 hospital visits in New York State over the period 2007–2013. The authors highlighted a significant, although moderate, increase of up to 2% in cardiovascular hospitalizations in the days following a pollution peak per each 5–7 µg/µm^3^ increment in PM_2.5_ concentrations [97]. These data are illustrative of the results from large national datasets in North America and Europe, reporting that short-term variations in PM_2.5_ concentrations are associated with 0.5–1% increases in total cardiovascular hospitalizations per each 10 μg/m^3^ across lags of 1–5 days [98–102]. Interestingly, in a study conducted in the contemporary US, where > 90% of days had daily PM_2.5_ concentrations < 15 μg/m^3^, Sun et al*.* still found statistically significant increases in cardiovascular hospitalizations and emergency department (ED) visits with higher daily PM_2.5_ indicating a risk well below current WHO guideline levels [103]. In Europe, Italian national analyses using flexible concentration–response functions likewise show no clear threshold down to relatively low PM levels, with significant associations for hospitalizations related to total cardiovascular diseases, stroke, ischemic heart disease and heart failure [100]. These findings strongly support the absence of a safe threshold for acute CVD morbidity. Similar results, are reported for short-term exposure to NO, which emerges as a robust predictor of cardiovascular admissions in several national and regional studies in Europe [104–108] and North America [109], proving at least as consistent as PM for coronary and arrhythmic endpoints. Of note, associations with cardiovascular admissions for O_3_ are less consistent than for PM/NO_2_. On the one hand, positive associations with acute myocardial infarctions (AMI), pulmonary heart disease, or broader cardiovascular hospitalizations have been found in warm season North American analyses conducted in the 1990s [110, 111] and Spanish national burden estimates attribute some CVD admissions to O_3_, particularly in the context of heat [105]. Conversely, many studies report null or even inverse O_3_–cardiovascular associations, reflecting negative correlations with traffic pollutants and seasonal confounding [112–114].

##### Respiratory admissions

Across North America, Western Europe, Australia, and high-income Asia, numerous time-series and case-crossover studies report positive associations between day-to-day variations in pollution and respiratory ED visits and hospital admissions for acute respiratory causes.

Using the SPARCS database, Croft et al*.* found an excess of emergency visits for influenza and pneumonia in the days following a pollution peak. This increase in ED visits was observed as early as 48 h post-peak, with a maximum 4% excess of consultations for influenza at day 6 per each 5–7 µg/µm^3^ increment in PM_2.5_ concentrations [115]. These results are in line with the literature, demonstrating that pneumonia hospitalizations and ED visits are associated with variations in exposure to PM and gases [111, 116–121]. Interestingly, studies that adjust directly for viral activity and pollen still report independent pollution effects [122–124], consistent with pollution acting as a co-factor that lowers host defenses or worsens infection severity rather than serving as the sole cause of infection.

Furthermore, a large body of literature on short-term air pollution focuses on the relationship with COPD exacerbations. European and Asian studies on hospital and ED outcomes show clear associations between daily NO_2_ and PM levels and both ED visits and hospital admissions for COPD exacerbations, highlighting a 1–3% increase per 10 μg/m^3^ increments in PM or NO_2_ with even higher risks observed in very elderly cohorts [121, 125–130]. Moreover, children emerge as the most sensitive subgroup regarding the short-term effects of air pollution on asthma (ED visits and hospitalizations). Large-scale US and CDC-based studies [131–135], spatially resolved NO_2_ analyses in England [136], and multi-pollutant studies in Europe and Asia [121, 124, 125, 137] all report larger relative risks in children than in adults [138, 139].

Notably, wildfires are among the specific contexts most strongly associated with acute respiratory events. This is underscored by a study analyzing 127,000,000 ED visits from the California health system over the period 2006–2017, which evaluated consultation frequencies within seven days of peak PM_2.5_ levels. The authors highlighted an excess of ED visits for respiratory reasons, averaging 10% for moderate intensity PM_2.5_ peaks between 10 and 25 µg/m^3^. Emergency visits for respiratory reasons increased by 30% on average for more significant peaks, above 50 µg/m^3^, and even exceeded 50% for acute exacerbations of asthma and COPD [140]. More recent studies have complemented these results, particularly showing the impact of PM_2.5_ from wildfire smoke, associated with an increase in hospitalizations for acute exacerbations of asthma, and to a lesser extent, COPD [141].

Altogether, these data strongly demonstrate that across diverse high-income settings, short-term increases in PM‚ NO_2_, and O_3_ are consistently associated with higher risks of respiratory ED visits and hospital admissions. Interestingly, these effects are detectable at pollutant levels below current regulatory standards in high-income countries, and the aggregated evidence supports no clear threshold.

##### Critical illness

Overall, the literature assessing the link between critical illness and short-term air pollution supports both a triggering effect of short-term particulate exposure on ICU admission rates, and a modulating effect of recent ambient exposure on early ICU severity, particularly through respiratory and cardiopulmonary pathways. A detailed summary of the main studies addressing this question is provided in Table 1.

**Table 1:** Short‑term ambient air pollution and critical illness studies

Authors (date)	Pollutants and shortterm window	Outcome/disease	Population and setting	Sample size	Design	Age group	Key shortterm effect sizes	Study quality
Groves et al. (2020)	NO_2_, PM_10_, PM_2.5_; short‑term daily exposure around admission, lags 0–2 days	Emergency ICU admissions for cardiorespiratory disease, stroke, sepsis; 30day mortality after ICU admission	ICUs in Australia and New Zealand (ANZICS‑APD), 2008–2016	46,965 ICU admissions	Ecologic (timestratified casecrossover/timeseries)	Adults	No significant association with ICU admission counts; 30‑day mortality increased with PM_2.5_: RR 1.18 (95% CI 1.02–1.37) per 10 µg/m³, stronger in ≥65 years (RR 1.33)	High (large multicenter registry, validated ICU outcomes, modern case‑crossover, good confounder control; null for incidence but robust for mortality)
Sorensen et al. (2021)	Wildfirerelated PM_2.5_; lags 0–5 days	Allcause ICU admissions	USA hospitals, 2006–2015 (≈15–20% of U.S. ICU admissions)	309,293 ICU admissions	Ecologic (timestratified casecrossover, distributed lag conditional Poisson)	Predominantly adults	+2.7% ICU admissions per 10 µg/m³ wildfire PM_2.5_ (95% CI 1.3–4.1) at lag 5; predicted ICU bed use up to 131% of baseline during a severe 7‑day, 120 µg/m³ smoke wave	High (national multi‑hospital data, strong design, explicit ICU endpoint, careful lag modeling)
Rublee et al. (2020)	Dust storm events; controlled for PM_2.5_, O_3_, meteorology; lags 0–5 days	All‑cause ICU admissions and respiratory ICU admissions	USA hospitals, 2000–2015 (≈15–20% of U.S. ICU admissions)	33,679 ICU admissions	Ecologic (timestratified casecrossover, distributed lag conditional Poisson)	Predominantly adults	All‑cause ICU admissions: +4.8% (95% CI 0.4–9.4) on dust‑storm day (lag 0). Respiratory ICU admissions: +9.2% (1.8–17) at lag 0; +7.5% (0.3–15.2) at lag 5	High (national multi‑hospital data, robust time‑stratified case‑crossover with distributed lags; main limitations: event‑based binary exposure and aggregate‑level data)
De Weerdt et al. (2020)	PM_2.5_, PM_10_, NO_2_, BC; lags 0–10 days	Duration of invasive mechanical ventilation	Patients ventilated ≥48 h on admission, Antwerp Univ. Hospital ICU (Belgium),	2,003 ICU patients	Patient‑level retrospective cohort with modeled exposures	Adults	Per 1.2 µg/m³ (1 IQR) increase in BC over prior 10 days: +12.4% ventilation duration (95% CI 4.7–20.7); per PM_2.5_, PM_10_, NO_2_ IQR increase → +7.8–8% duration ventilation	High (fine‑scale exposure modeling, ICU‑level outcome, appropriate distribution lag modeling and adjustment)
Yüksel Yavuz et al. (2025)	Monthly mean PM₁₀ and SO₂, with a 1‑month lag for cardiovascular outcomes	ICU admissions for pulmonary and cardiovascular diseases; inhospital mortality	Single tertiary hospital (Middle East), 2012–2019	6,112 ICU admissions; analyses restricted to 227 pulmonary and 344 cardiovascular admissions	Ecologic (monthly timeseries, multivariable regression	Adults	Higher PM₁₀ associated with pulmonary ICU admissions (*β* = 0.017; 95% CI 0.003–0.031) and cardiovascular ICU admissions at 1‑month lag (β = 0.018; 95% CI 0.002–0.034). Effect scale is per 1 µg/m³ increase in monthly PM₁₀ (ecologic).	Moderate (ICU‑specific but small sample size, monthly—not truly “classic” short‑term—exposure; single center)
Reilly et al. (2023)	O₃, NO₂, SO₂, CO, PM_2.5_, PM₁₀; 3day lag	ARDS incidence within 6 days in sepsis	Patients admitted with sepsis, academic center ICU (USA), 2008-2018	1,858 patients with sepsis; 754 (41%) developed ARDS	Patientlevel prospective ICU cohort, multivariable logistic regression	Adults	Higher pollutants concentrations (75th vs 25th percentile) significantly associated with ARDS, for 3-days NO₂ (OR1.37 (95% CI 1.10–1.71)), SO₂ (OR 1.31 (95% CI 1.06–1.63)), PM2.5 (OR 1.07 (95% CI 0.90–1.28))	High (prospective design, detailed phenotyping & confounder control)
Im et al. (2020)	PM₁₀, SO₂, CO, O₃; measured on the ICU admission day based on residential postcode	90day mortality overall; pulmonary 90day mortality in COPD	Singlecenter ICU cohort Tertiary academic hospital ICU (Korea), 2015–2016	10,276 ICU admissions (8,752 patients)	Patientlevel retrospective ICU cohort, Cox regression	Adults	Overall: no significant association between pre‑ICU pollution and 90‑day mortality. In COPD patients, O₃ HR 1.04 (95% CI 1.01–1.08) per 0.001 ppm increase, CO HR 5.99 (95% CI 1.51–23.83) per 1 ppm increase for pulmonary‑related 90‑day mortality	Moderate (good adjustment for ICU covariates; but small COPD subgroup, very high increment per 1 ppm, outcome far downstream of exposure)
Reilly et al. (2019)	O₃, NO₂, SO₂, CO, PM_2.5_; 3day lag	ARDS incidence within 6 days of severe trauma	Severely injured ICU patients (ISS>15) in prospective trauma cohort (USA), 2005-2015	996 patients; 243 (24%) developed ARDS	Patientlevel prospective single-center ICU cohort; multivariable logistic regression	≥13 years	Short‑term (3‑day) exposures largely null, except non‑linear association for SO₂ (OR 1.78 (1.26–2.50) for 75th vs 25th percentile).	High (good individual phenotyping, good confounder control including APACHE III and ISS; short‑term signal weak, highlighting importance of chronic exposure)
Rush et al. (2018)	O₃, 8h concentrations on a daily basis	Hospital mortality in sepsis	Nationwide inpatient sample (NIS, USA); All patients from 2011; hospitalized adults with sepsis	444,928 patients with sepsis	Retrospective national administrative cohort, patientlevel; hospital arealevel exposure	Adults	After adjustment for sepsis severity, higher O₃ associated with increased mortality: per 0.01 ppm increase OR 1.04 O₃ (95% CI 1.03-1.05) overall, 1.06 (95% CI 1.04-1.08) in pneumonia, 1.02 (95% CI 1.01-1.03) in non-pneumonia.	Moderate (very large sample size; outcome proximate to ICU; limited by coarse exposure, administrative assessment of severity control, no ICU‑specific mortality/length of stay)
Cui et al. (2023)	PM_2.5_, PM₁₀, O₃, SO₂; daily averages before and during ICU stay; lags 0–7 day	VAP in ventilated cardiac surgery patients	Pediatric cardiac surgery ICU (China), 2013–2020	1,755 children; 348 VAP cases	Patientlevel single-center pediatric ICU cohort	Pediatric	Short‑term increase in PM_2.5_ (and some other pollutants) associated with higher VAP risk with delayed peaks over several days; precise RR/ORs depend on lag and concentration; overall 10–20% higher risk at upper quantiles	Moderate–High (good DLNM modeling and clear ICU outcome; pediatric, disease‑specific, limits generalizability to adults)

Studies are listed according to their relevance to the topic. Study quality was categorized as follows: High in case of large or well‑characterized cohort/registry, ICU or critical‑illness outcomes defined with standard criteria, short‑term exposure assessed with reasonable spatial resolution and appropriate lag models (e.g., DLNM, time‑stratified case‑crossover), confounding by temperature, season, and key clinical factors substantially addressed; Moderate in case of single‑center, smaller samples, or coarser exposure metrics (e.g., monthly averages), ICU/critical‑illness outcome clear but restricted to specific diagnoses or age groups, confounding handled but with some limitations (e.g., possible residual confounding, ecologic lag structures). ARDS, acute respiratory distress syndrome; DLNM, distributed log non-linear model; ICU, intensive care unit; ISS, injury severity score; VAP, ventilator associated pneumonia

A study conducted in the USA over the period 2000–2015 examined the impact of sandstorms, associated with high ambient levels of PM_10_ and PM_2.5_, on ICU admission frequency [142]. This study found an average increase of nearly 10% in ICU admissions for respiratory reasons on sandstorm days, after controlling for temperature and seasonality. In another study, involving a very large cohort of more than 700,000 ICU admissions in the USA, the authors found a significant increase in ICU admissions within 3–5 days following a pollution peak resulting from wildfires [143]. Furthermore, the study by Silverman and Ito is, to date, the main work evaluating the effect of PM_2.5_ and O_3_ peaks on the risk of ICU admission for acute asthma exacerbation [144]. In this study involving more than 70,000 hospital stays for asthma attacks, including 6,000 ICU admissions, the authors confirmed the association between particulate and ozone pollution peaks related to warm weather patterns on one hand, and hospital admissions for asthma attacks within 24 h on the other. However, regarding ICU admissions across all age groups, only a non-significant trend was found for PM_2.5_ peaks (RR 1.05 (0.99–1.11) per 12 µg/m^3^ increase), mainly driven by the pediatric patient subgroup. These analyses directly quantify all-cause ICU surge in the general hospital population during high-intensity particulate events.

Excluding extreme events, in an analysis of nearly 50,000 ICU admission for cardiorespiratory events, stroke and sepsis admissions from the ANZICS database by Groves et al. reported a significant association between higher short-term PM_2.5_ levels and an increased ICU admission burden [145]. In addition, Yüksel Yavuz report using monthly PM_10_ data, that higher levels were associated with increased pulmonary ICU admissions as well as cardiovascular ICU admissions at a 1-month lag [146]. Together, these findings suggest that even beyond extreme events, variations in urban PM are measurably linked to ICU admission incidence.

Regarding outcomes in ICU patients, De Weerdt, *el al.* modeled residential PM_2.5_, NO_2_ and black carbon at home address over the 10 days preceding ICU admission in 2,003 adults ventilated for at least 48 h, and found that higher pre-ICU exposure over specific lags was associated with prolonged duration of mechanical ventilation, independent of baseline covariates. This is one of the clearest demonstrations that recent ambient exposure prior to critical illness onset worsens early ICU course, even after conditioning on ICU admission [147]. In addition, two studies by Reilly et al. found an association between elevated levels of PM_2.5_, NO_2_, and SO_2_ over the preceding five days and the risk of developing ARDS in patients admitted for polytrauma or sepsis [148, 149]. However, these studies suggest that short-term triggers are less dominant than chronic or subacute exposure for trauma-related ARDS, whereas short-term effects may be more pronounced in infection-mediated pathways, such as sepsis and pneumonia. Similar results were also observed for the risk of developing ARDS from all causes in a cohort of ICU patients in China [150].

As for late complications, Cui et al. [151] demonstrated that higher short-term PM exposure before and during ICU stay increased the risk of ventilator-associated pneumonia (VAP) in pediatric cardiac surgery patients. These findings, which revealed clear distributed lag effects, suggest that ambient PM in the peri-ICU period can directly influence the development of nosocomial complications.

Finally, in a general adult ICU cohort in Korea, pre-event exposure to four pollutants showed no overall association with 90-day mortality [152]. However, among patients with pre-existing COPD, higher O_3_ levels were linked to increased pulmonary-related 90-day mortality [99]. This suggests that susceptible patients with COPD may experience pollution-related clinical worsening that extends well beyond the initial ICU stay.

From the intensivist’s perspective, short-term air pollution therefore appears to be associated with an influx of more numerous and more severely ill patients admitted for respiratory and cardiovascular reasons. This pattern aligns with biological expectations: acute pollution spikes can precipitate critical decompensations and prime the lungs and systemic inflammatory milieu, thereby exacerbating early organ dysfunction and infection risk once critical illness is established. However, epidemiological data remain fragmented, and the actual impact on the pressure exerted on ICU bed capacity remains to be fully elucidated.

#### Chronic exposure

The impact of chronic exposure to atmospheric pollutants on human health has been the subject of numerous studies. Among these, the work of the ELAPSE consortium, involving nearly 400,000 subjects followed from 1990 to 2018 across 11 cohorts from 22 European countries, is particularly noteworthy [5]. The authors reported a significant association between PM_2.5_ and NO_2_ and the incidence of deaths from natural causes. The hazard ratio for the excess risk of death from natural cause was measured at an average of 1.4 for PM_2.5_ levels of around 10 µm/m^3^—equivalent to what is found in a large European city [153], compared to a reference level of 3 µg/m^3^.

##### Cardiovascular admissions

The European ESCAPE pooled cohorts [154], North American studies [155–157], Scandinavian source-specific cohorts [158–161], classic US female cohorts [162–164], and Kaiser California cohort [165] all demonstrate an elevated risk of first AMI or acute coronary events per unit increase in PM_2.5_ and/or NO_2_. However, findings appear more nuanced in the European ELAPSE multi-cohort, where risk is significantly increased by high NO_2_ levels but shows no such association with PM_2.5_ [5]. This association is also observed in stroke incidence, with risk consistently rising alongside PM_2.5_ and NO_2_ concentrations. This is supported by data from North America, Europe, and Korea [166–176], including analysis of pollutant levels below current air quality standards. Furthermore, research on the linkage between atrial fibrillation and stroke indicates that PM_2.5_ and NO_2_ increase both incidence and progression in Ontario and UK Biobank cohorts [177, 178]. However, some Japanese cohorts show weaker or null associations for stroke incidence and mortality [179, 180], contrasting with previously reported findings; the reasons for this discrepancy (exposure metrics, stroke subtypes, confounding, or statistical power) remain to be fully clarified. On the other hand, large administrative and cohort studies consistently find higher incidence of heart failure and atrial fibrillation with higher long-term PM_2.5_ and NO_2_ [156, 166, 168, 172, 173, 178, 181–184].

Notably, these studies found a robust association between higher PM_2.5_, NO_2_, and O_3_ levels and first hospitalizations for AMI, stroke, heart failure, atrial fibrillation, and broader cardiovascular diseases events. This strengthens the evidence that acute clinical events severe enough to require hospitalization are pollution-related. Finally, among patients with a prior AMI or stroke, long-term exposure to PM_2.5_ and NO_2_ increases the risk of recurrent hospitalizations for and post-event cardiovascular-related mortality. This suggests that chronic exposure impacts not only disease onset but also disease progression and vulnerability to secondary events [177, 185, 186].

##### Respiratory admissions

Large-scale studies in the USA, the UK, Scotland, Spain, and Australia consistently associate higher PM_2.5_, NO_2_, O_3_, SO_2_ and black carbon levels with increased hospital admissions for pneumonia and acute lower respiratory infections (ALRI), particularly among individuals over 65 [166, 174, 187–191]. Notably, a US cohort of 60 million subjects with data collected between 2000 and 2016 identified an excess risk of pneumonia hospitalization linked to high levels of PM_2.5_ and O_3_; this risk was also observed, albeit to a lesser extent, for NO_2_ exposure [166]. Furthermore, data from the Danish Nurse Cohort indicate that 3-year moving means of PM_2.5_, NO_2_, and black carbon are linked not only to the first occurrence of ALRI but also to recurrent episodes requiring hospital contact [192].

Beyond acute infections, long-term exposure to PM_2.5_, NO_2_, O3 and black carbon is a major driver of chronic obstructive pulmonary disease (COPD) and asthma morbidity. Data from US Medicare, English, and Scottish cohorts, and from the ELAPSE European multi-cohort, demonstrate that hospital admissions for COPD and asthma increase significantly with chronic pollutant exposure [5, 166, 174, 193, 194]. Similarly, studies in Sydney and Perth link these pollutants to acute exacerbations in older adults [188, 189]. Notably, large-scale longitudinal studies indicate that chronic pollution not only triggers acute episodes but also increases the overall incidence of obstructive lung diseases [193, 195, 196]. These findings suggest that long-term exposure expands the pool of vulnerable individuals with pre-existing lung disease, thereby increasing susceptibility to severe, pollution-induced acute events.

#### Critical illness

Long-term exposure to ambient PM_2.5_, NO_2_, and O_3_ in high-income countries is consistently associated with a higher risk of developing ICU-level acute respiratory failure, particularly ARDS. Furthermore, such exposure is linked to increased severity of critical illness, as evidenced by higher rates of ICU admission, the need for invasive support, and mortality. A detailed summary of the main studies addressing this question is provided in Table 2.

**Table 2:** Long-term Ambient Air Pollution and Critical Illness Studies

Authors (date)	Pollutants and long-term window	Outcome/disease	Population and setting	Sample size	Design	Age group	Key short term effect sizes	Study quality
Gutman et al*.* (2022)	NO_2_, PM_10_, PM_2.5_, O₃; 1-year average before event (regional models)	ICU admission for ARDS; 90-day mortality	Patients admitted with ARDS, Provence-Alpes-Côte-d’Azur, France (PMSI), 2016–2018	4,733 ICU patients with ARDS	Patient-level ICU cohort from administrative data; exposure at area level	Adults	PM_2.5_, 1-year: IRR 1.21 (95% CI 1.15–1.39) per 1 µg/m^3^ for ARDS incidence and OR 1.096 (95% CI, 1.001–1.201) for 90-day mortality. Similar trends observed for NO_2_/PM_10_ and ARDS incidence	Moderate (large, population‑based ICU cohort; prognosis limited by administrative severity data, area‑level exposure)
Ware et al*.* (2016)	O₃, NO₂, SO₂, PM_2.5_, PM₁₀; 3-year average at nearest monitor (≤ 50 km)	Development of ARDS in ICU among patients with risk factors	Critically ill medical/surgical/trauma patients, Vanderbilt ICU (USA)	1,558 ICU patients	Prospective patient-level ICU cohort	Adults	O₃ 3-years: ARDS incidence 42% (quartile 3) vs. 28% (quartile 1), OR 2.54 (95% CI 1.46–3.50); Significant interaction with smoking status; NO₂ associated but not independent; SO₂, PM_2.5_, PM₁₀ not associated	High (good ICU phenotyping, long‑term exposure, APACHE‑adjusted; exposure coarseness and collinearity remain)
Reilly et al*.* (2019)	O₃, NO₂, SO₂, CO, PM_2.5_; 3-year average; monitors ≤ 50 km of home	ARDS incidence within 6 days of severe trauma	Severely injured ICU patients (ISS > 15) in prospective trauma cohort (USA), 2005–2015	996 patients; 243 (24%) developed ARDS	Patient-level prospective single-center ICU cohort; multivariable logistic regression	≥13 years	Long-term higher ambient pollutants levels (75th vs 25th percentile) associated with elevated ARDS risk: O₃ (OR 1.44 (IC 95% 1.12–1.86)), NO₂ (OR 2.39 (IC 95% 1.72–3.33)), SO₂ (OR 3.56 (IC 95% 2.40–5.28), CO (CO 1.92 (IC 95% 1.47–2.53)), PM_2.5_ (OR 3.58 (IC 95% 2.40–5.34) each independently	High (good individual phenotyping, good confounder control including APACHE III and ISS; some exposure misclassification & multicollinearity)
Reilly et al. (2023)	O₃, NO₂, PM_2.5_; 5-year average; monitors ≤ 50 km of home	ARDS incidence within 6 days in sepsis	Patients admitted with sepsis, academic center ICU (USA), 2008–2018	1,858 patients with sepsis; 754 (41%) developed ARDS	Patient-level prospective ICU cohort, multivariable logistic regression	Adults	Long-term higher ambient pollutants levels (75th vs 25th percentile) associated with elevated ARDS risk: NO₂ (OR 1.36 (IC 95% 1.06–1.74)), SO₂ (OR 1.43 (IC 95% 1.16–1.77), PM_2.5_ (OR 1.21 (IC 95% 1.04–1.41) each independently	High (prospective design, detailed phenotyping & confounder control)
Rush et al*.* (2017)	O₃ (list of top-level cities vs others according to 8 h concentrations on a daily basis), PM_2.5_ (annual mean) (city/county level);	Hospital mortality in ventilated ARDS patients	Nationwide inpatient sample (NIS, USA); All patients from 2011; hospitalized adults with ARDS receiving invasive ventilation	93,950 patients with ARDS	Retrospective national admin cohort, patient-level; area-level exposure	Adults	In the 15 top-O_3_ cities vs others: unadjusted mortality 34.9% vs 30.8%; adjusted OR for hospital mortality 1.11 (95% CI 1.08–1.15). No effect for PM_2.5_	Moderate (very large sample size; outcome proximate to ICU; limited by coarse exposure, administrative assessment of severity control, no ICU‑specific mortality/length of stay)
Rush et al*.* (2018)	PM_2.5_, annual (county-level data)	Hospital mortality in sepsis	Nationwide inpatient sample (NIS, USA); All patients from 2011; hospitalized adults with sepsis	444,928 patients with sepsis	Retrospective national admin cohort, patient-level; hospital area-level exposure	Adults	No effect for PM_2.5_	Moderate (very large sample size; outcome proximate to ICU; limited by coarse exposure, administrative assessment of severity control, no ICU‑specific mortality/length of stay)
Rhee et al*.* (2019)	PM_2.5_, O₃; annual ZIP-code level averages	Hospital admissions for ARDS	Patients admitted with ARDS (Medicare, USA); 2000–2012	1,164,784 patients with ARDS admissions	Nationwide patient-level cohort; area-level exposure	≥ 65 years	Increase in annual ARDS admission rate: per each 1 µg/m^3^ PM_2.5 i_ncrease: + 0.72% (95% CI 0.62–0.82); per each 1 ppb O₃ increase: + 0.15% (95% CI 0.08–0.22); stronger effects at low pollution levels	Moderate (very large cohort, good exposure surfaces; residual confounding possible)

Studies are listed according to their relevance to the topic. Study quality was categorized as follows: High in case of large or well‑characterized cohort/registry, ICU or critical‑illness outcomes defined with standard criteria, short‑term exposure assessed with reasonable spatial resolution and appropriate lag models (e.g., DLNM, time‑stratified case‑crossover), confounding by temperature, season, and key clinical factors substantially addressed; Moderate in case of single‑center, smaller samples, or coarser exposure metrics (e.g., monthly averages), ICU/critical‑illness outcome clear but restricted to specific diagnoses or age groups, confounding handled but with some limitations (e.g., possible residual confounding, ecologic lag structures). ARDS: acute respiratory distress syndrome, ICU: intensive care unit, ISS: injury severity score

Long-term exposure to atmospheric pollutants serves as a significant “gate” to critical illness, particularly through the development of (ARDS). Research from the VALID and Penn trauma cohorts consistently demonstrates that multi-year exposure to O_3_, NO_2_ and PM_2.5_ is strongly and independently associated with ARDS risk in the ICU, regardless of acute severity or comorbidities [148, 197]. Notably, the VALID cohort showed an OR of 2.5 for ARDS when comparing the highest versus lowest O_3._ In the Penn trauma cohort, which included over 1,000 polytrauma patients, Reilly et al*.* identified a near-linear relationship between chronic exposure to PM_2.5_, NO_2_, and SO_2_ and the risk of developing ARDS within seven days of admission. This risk escalated dramatically from 5% for an average PM_2.5_ level of 10 µg/m^3^ to 40% when levels reached 15 µg/m^3^. Similar results were more recently observed in a cohort of 1,800 septic patients, where high chronic exposure to PM_2.5_, NO_2_, and SO_2_ increased ARDS risk by day 6. These clinical outcomes are supported by biological markers; pollutant exposure in septic patients was associated with elevated circulating levels of inflammatory markers, specifically Interleukin-1 receptor antagonist (IL-1RA) and soluble tumor necrosis factor receptor 1 (sTNFR1), indicating a heightened systemic inflammatory state.

Among US Medicare beneficiaries over 65, higher annual PM_2.5_ and O_3_ levels at the ZIP-code level are associated with higher rates of hospital admission for ARDS [198]. Similarly, a French study by Gutman et al*.* involving nearly 5,000 ICU patients in the Mediterranean region, investigated the association between the incidence and severity of all causes ARDS and exposure to atmospheric pollutants [199]. Their results highlighted a significant association between ARDS incidence and long-term exposure to PM_2.5_ and NO_2_. Furthermore, the authors identified increased mortality among ARDS patients who had chronic exposure to high PM_2.5_ in the years preceding ICU admission.

Beyond increasing the risk of admission, long-term exposure to pollutants significantly worsens survival outcomes for critically ill patients. Analysis of the US Nationwide Inpatient Sample (NIS) revealed that mechanically ventilated ARDS patients in cities with high O_3_ levels face higher hospital mortality (adjusted OR 1.11) compared to those in lower-exposure areas [200]. These findings are mirrored in France by the study from Gutman et al*.* which reported an association between increased 90-day mortality and greater PM_2.5_ exposure among ARDS patients [199]. The impact extends to septic patients; higher chronic exposure to both O_3_ and PM_2.5_ is associated with increased in-hospital sepsis mortality, even after adjusting for validated clinical severity scores [201]. While these signals are consistent across different healthcare systems, it should be noted that prognostic analyses often rely on regional exposure metrics and administrative covariates, highlighting the need for increased precision in exposure modelling.

Finally, the specific impact of chronic pollutant exposure on the incidence and severity of SARS-CoV-2 infections has been evaluated in numerous studies [202], the majority of which suggest a probable relationship between COVID-19 incidence and outcomes and long-term exposure to atmospheric pollutants.

## Limitations, critical gaps, and perspectives in air pollution and critical care research

While epidemiological evidence consistently links exposure to major pollutants, notably PM_2.5_, O_3_, and NO_2_ with increased frequency and severity of acute illnesses, current research lacks the granularity needed to fully quantify the burden on ICU. To move beyond simple association and toward a comprehensive understanding of the pollution-to-critical-illness pathway, future research must address several critical areas.

### Clinical trajectories and severity metrics

Current literature focuses heavily on admission rates and basic outcomes, such as mortality or ARDS incidence. There is a significant need to incorporate high-resolution physiological data to better understand patient trajectories. Future studies should utilize validated scores (e.g., SOFA, APACHE, or SAPS) to track multi-organ failure. Furthermore, research must link long-term exposure to specific interventions, including the duration of mechanical ventilation and the requirement for vasopressors, renal replacement therapy, or ECMO.

### Standardization

The field is currently hampered by heterogeneity, which complicates meta-analyses. Improvement requires standardized exposure windows to resolve the inconsistent use of 1, 3, or 5-year averages for long-term exposure and 1, 3, 7-day windows for short-term effects. In addition, data sources must be improved and homogenized, by leveraging national ICU registries through robust case-crossover or time-series designs, moving away from highly selected or disease-specific cohorts. Finally, the field should rely on harmonized definitions, creating a unified framework for what constitutes "critical illness" and "ICU outcomes" within environmental health research.

### Causal modelling and bias mitigation

To establish a definitive link, researchers must address the complex causal structures of pollution-related illness. This can be addressed by expanding translational research, which should focus on refining preclinical models to better reproduce “real-life” exposure to air pollution. This includes accounting for dosage, chemical composition, and timing through an integrated approach that considers the entire exposome. It can be achieved too by mediation analysis, which is critical to determine whether pollution-related mortality is mediated by baseline frailty (pre-existing conditions), a direct increase in acute physiological severity, or other environmental confounders (e.g. meteorologic or epidemic seasonality). Moreover, because many studies only analyzed patients already admitted to the ICU, "conditioning on the index event" can introduce significant collider bias. Advanced causal modelling is required to map the entire pathway from exposure to acute event, through to hospitalization and eventual ICU outcomes. Similarly, the impact of continued air pollution exposure after hospital and ICU admission remains a critical are for further investigation.

## Conclusion

In total, data from environmental observations suggest a future increase in situations associated with deteriorating air quality, particularly pollution spikes. This reflection must be conducted in a holistic manner, considering the future rise in respiratory diseases globally, resulting notably from rapid population growth in areas with degraded air quality [203]. These situations should lead to the integration of these events into the organization of our healthcare ecosystem. The additional influx of patients to hospitals in the context of air pollution appears to potentially impact critical care services in two ways. On the one hand, it could cause a mechanical increase in admissions to intensive care units, and on the other, it could exert additional pressure on the availability of beds in recovery units.

Beyond these organizational aspects, the direct impact of pollution on the reasons for patients being admitted to intensive care should prompt us to consider the existence of a phenotype in individuals, for whom exposure to pollutants could trigger exacerbations leading to hospitalization.

Finally, the impacts of pollution on the trajectory of patients once admitted to intensive care remain poorly understood. Data suggesting impaired immune response quality raise concerns about the possibility of an increased frequency of complications associated with intensive care, starting with infections.

All the aforementioned challenges highlight the need for a reflection that goes beyond the narrow scope of human health, also preserving the balance of ecosystems and environmental conditions. Such an approach, inspired by the "One Health" concept, seems capable of improving the health of populations, with a positive impact on intensive care patients [204].

Epidemiological and laboratory evaluation tools are continuously improving and should enable a better understanding in the near future of the exact implications of these findings for intensivists.

## Data Availability

Not applicable.
